# Modeling the ferrochelatase c.315-48C modifier mutation for erythropoietic protoporphyria (EPP) in mice

**DOI:** 10.1242/dmm.027755

**Published:** 2017-03-01

**Authors:** Jasmin Barman-Aksözen, Paulina C´wiek, Vijay B. Bansode, Frank Koentgen, Judith Trüb, Pawel Pelczar, Paolo Cinelli, Xiaoye Schneider-Yin, Daniel Schümperli, Elisabeth I. Minder

**Affiliations:** 1 Institute of Laboratory Medicine, Municipal Hospital Triemli, Zürich 8063, Switzerland; 2 Institute of Cell Biology, University of Bern, Bern 3012, Switzerland; 3 Ozgene Pty Ltd, Bentley DC, WA 6983, Australia; 4 Center for Transgenic Models, University of Basel, Basel 4002, Switzerland; 5 Division of Trauma Surgery, University Hospital Zürich, Zürich 8091, Switzerland; 6 Porphyria Outpatient Clinics, Municipal Hospital Triemli, Zürich 8063, Switzerland

**Keywords:** Liver dysfunction, Mouse model, Photosensitivity, Protoporphyrin IX (PPIX), Rare disease, Splicing defect

## Abstract

Erythropoietic protoporphyria (EPP) is caused by deficiency of ferrochelatase (FECH), which incorporates iron into protoporphyrin IX (PPIX) to form heme. Excitation of accumulated PPIX by light generates oxygen radicals that evoke excessive pain and, after longer light exposure, cause ulcerations in exposed skin areas of individuals with EPP. Moreover, ∼5% of the patients develop a liver dysfunction as a result of PPIX accumulation. Most patients (∼97%) have a severe *FECH* mutation (Mut) *in trans* to an intronic polymorphism (c.315-48C), which reduces ferrochelatase synthesis by stimulating the use of an aberrant 3′ splice site 63 nt upstream of the normal site for exon 4. In contrast, with the predominant c.315-48T allele, the correct splice site is mostly used, and individuals with a T/Mut genotype do not develop EPP symptoms. Thus, the C allele is a potential target for therapeutic approaches that modify this splicing decision. To provide a model for pre-clinical studies of such approaches, we engineered a mouse containing a partly humanized *Fech* gene with the c.315-48C polymorphism. F1 hybrids obtained by crossing these mice with another inbred line carrying a severe *Fech* mutation (named m1Pas) show a very strong EPP phenotype that includes elevated PPIX in the blood, enlargement of liver and spleen, anemia, as well as strong pain reactions and skin lesions after a short period of light exposure. In addition to the expected use of the aberrant splice site, the mice also show a strong skipping of the partly humanized exon 3. This will limit the use of this model for certain applications and illustrates that engineering of a hybrid gene may have unforeseeable consequences on its splicing.

## Abstract (precis)

**Summary:** A new mouse model reproduces the predominant genetic disposition of patients affected by erythropoietic protoporphyria, a rare disease associated with extreme pain after light exposure.

## INTRODUCTION

Erythropoietic protoporphyria (EPP; MIM 177000) is a rare hereditary disorder of heme biosynthesis. Its most frequent cause is a partial deficiency in ferrochelatase (FECH; EC 4.99.1.1), the last enzyme of the heme biosynthesis pathway. EPP can also be due to an increase in δ-aminolevulinate synthase 2 (ALAS2; EC 2.3.1.37), the first and rate-limiting enzyme of the pathway. The overall frequency of this latter change could be between 2 and 10%, as some reports have indicated (Balwani et al., 2013; Whatley et al., 2010). Both types of alteration lead to the excessive accumulation of protoporphyrin IX (PPIX), a heme ring that has not yet been complexed with iron (Anderson et al., 2001). Activation of PPIX by light in the visible wavelength range induces the formation of singlet oxygen. If excessive amounts of PPIX accumulate in red blood cells (RBCs) and plasma of skin micro-vessels, as is the case in EPP patients, severe phototoxic skin reactions occur within a few minutes of exposure to sunlight or intense artificial light. Depending on the intensity of light irradiation and the individual level of PPIX accumulation, excruciating and incapacitating pain results, lasting up to 2 weeks. This may be accompanied by additional signs such as edema, petechia, blisters and erosions. In severe cases, when the PPIX concentrations reach a level above 30 µmol/l RBCs, the liver can be affected by toxic effects of accumulated PPIX, which, ultimately, can lead to liver failure.

To date, there is no effective treatment for EPP. Most of the tested approaches aimed at preventing or reducing symptoms but had no or very limited success (Minder et al., 2009). In particular, the strong pain symptoms do not respond to any of the conventional analgesics (Thunell et al., 2000), even though there is a recent indication that antagonists of the TRPA1 and TRPV1 polymodal receptor channels may work (Babes et al., 2016). Recently, the α-melanocyte-stimulating hormone analog afamelanotide was shown to postpone the occurrence and reduce the severity of EPP symptoms by stimulating skin pigmentation (Biolcati et al., 2015; Harms et al., 2009; Langendonk et al., 2015), but this still is a purely symptomatic treatment. A treatment and eventually a cure for EPP can be achieved by bone marrow transplantation. However, because of the inherent severe immunological risks, experts recommend this procedure only in cases of recurrent life-threatening liver complications (Anstey and Hift, 2007; Minder et al., 2016; Wahlin et al., 2007). Gene therapy using a *FECH* cDNA expression construct has been proven to work experimentally in mice (Pawliuk et al., 1999; Richard et al., 2001), but has not been advanced to the clinical stage. Thus, there is a strong need for an efficient treatment of the underlying causes of EPP.

EPP can be caused by three different genetic aberrations (Lecha et al., 2009): (1) an activating mutation in *ALAS2*; (2) compound heterozygous or homozygous *FECH* mutations resulting in a recessive form; and (3) the combination of a strongly hypomorphic or null mutation of one *FECH* allele *in trans* to a C-polymorphism (instead of T) at position −48 with respect to the 3′ splice site of exon 4 (c.315-48C). This single nucleotide exchange leads to a lower expression of this allele by increasing the usage of an alternative 3′ splice site (ss) positioned 63 nucleotides upstream of the normal one (Gouya et al., 2006, 2002). The use of this alternative ss inserts 63 additional nucleotides into the RNA product. Two in-frame stop codons in this extra sequence prevent the formation of functional ferrochelatase and target the transcript to nonsense-mediated mRNA decay (NMD). This last type of genetic disposition is by far the most abundant, amounting to ∼97% of all *FECH*-related EPP cases.

Because of its high frequency among EPP patients, the aberrant splicing caused by the c.315-48C polymorphism is an attractive target for therapeutic interventions. Such a splicing modulation is conceivable with antisense oligonucleotides, genes for antisense RNAs introduced by gene therapy, or even small-molecule drugs selected to modulate a specific ss with high specificity (Nlend Nlend and Schümperli, 2012; Scotti and Swanson, 2016). In the case of EPP, recent studies showed that certain antisense oligonucleotides are able to shift the ss preference of *FECH* exon 4 from the alternative to the conventional ss, thereby enhancing the production of functional *FECH* mRNA and protein (Li et al., 2017; Oustric et al., 2014).

To develop such therapeutic approaches towards a clinical stage, appropriate animal models are required. A first mouse EPP model designated as m1Pas was described in 1991 (Boulechfar et al., 1993; Tutois et al., 1991). In this model, an EMS-induced missense mutation in *Fech* exon 3 produces a ferrochelatase enzyme with ∼6% residual activity. Another EPP mouse model based on deletion of exon 10 was described by Magness and Brenner (1999) and Magness et al. (2002) and was homozygously lethal. This exon 10 deletion was further investigated by Bloomer et al. (2008) in a high and low *Fech* expression background. They demonstrated that the expression level of the non-mutant allele determines the penetrance of the mutation in the heterozygous state. However, despite the fact that the PPIX level was increased in the low expression background, it was still much lower than in human EPP.

Although these models have provided useful information on genetic and pathophysiological aspects of EPP and can also be used to explore certain types of therapies, it is important to note that none of them mimics the human c.315-48C polymorphism that contributes to EPP in the vast majority of human EPP patients. Thus, in order to provide a model that can be used to develop and assess splicing modulation therapies for EPP, we engineered mice with a partly humanized *Fech* gene containing the c.315-48C polymorphism. By crossing these mice with m1Pas mice, we are able to generate a genotype closely reflecting human EPP. These compound heterozygous mice show a very severe EPP phenotype that is reflected by an elevated prenatal mortality rate and impaired growth, as well as strong photosensitivity, anemia and liver symptoms. However, this strong phenotype is not only due to the aberrant splicing caused by c.315-48C, but also, to a large extent, to another type of mis-splicing – the skipping of exon 3. The implications of this finding for future uses of our model will be discussed.

## RESULTS

### Creation of a humanized c.315-48C *Fech* allele by homologous integration

To generate a mouse model reproducing the enhanced aberrant splicing caused by the human *FECH* c.315-48C polymorphism, we amplified by PCR a suitable region of DNA from Epstein–Barr virus (EBV)-transformed lymphoblasts of a non-porphyric human homozygous for the c.315-48C allele (Barman-Aksözen et al., 2013). The amplified 2.1 kb fragment starting in exon 3 and ending in intron 4 (near the end of exon 4) was produced with forward and reverse primers that introduced restriction sites for *Xba*I and *Sal*I, respectively. The *Xba*I-*Sal*I fragment was then cloned in pBluescript, and several clones were verified by DNA sequencing. For further work, we selected clone C22, which differs from the reference sequence (NT_025028) only in the length of two poly(C) and poly(T) sequences, both of which are located in intron 3 (Fig. S1). Clone C22 contains 13 C and 23 T nucleotides compared with 11 C and 24 T in the reference sequence. In our sequence data from EPP patients and non-porphyric individuals from Switzerland and Israel, the length of the poly(C) tract varies from 10 to 16 and that of poly(T) from 22 to 24 (Barman et al., 2009). Most importantly, the combination of 13 C and 23 T found in clone C22 is naturally present in several individuals of this cohort.

**Fig. 1.**
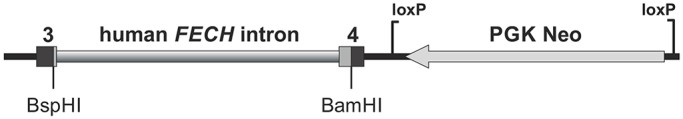
**Schematic view of the humanized *Fech* exon 3-4 region joined to a neomycin resistance cassette flanked by *loxP* sites.** The exon 3-4 fragment consists of mouse sequences from intron 2 up to the *Bsp*HI site in exon 3 and from the *Bam*HI site in exon 4 into intron 4 (Fig. S2). Mouse exon parts are shown as black boxes, human exon parts as dark gray ones. Note that the Neo cassette is in inverse orientation with respect to the *Fech* fragment. In the final targeting construct, this part of the assembly was joined to 5′ and 3′ homology regions of 6120 and 6006 bp, containing exons 2 and 5, respectively.

The *Fech* knock-in mouse line was then generated by Ozgene (Bentley, WA, Australia). The targeting vector for homologous recombination in mouse ES cells was generated by assembling in multiple steps: (1) A 1151 bp *Kpn*I-*Nhe*I fragment containing the interior of human *FECH* intron 3 was amplified by PCR from clone C22. (2) Two synthetic DNA fragments containing the hybrid mouse–human and human–mouse exons 3 and 4 were joined upstream and downstream of the human intron 3 fragment via the *Kpn*I and *Nhe*I sites, respectively. The mouse and human parts were fused at the *Bsp*HI and *Bam*HI sites of the two exons, respectively. The sequence of this part of the targeting vector is shown in Fig. S2. (3) A 1769 bp *Pac*I fragment containing a PGK promoter/neomycin resistance gene cassette flanked by *loxP* sites was added downstream of the hybrid exon 4 fragment. The neomycin resistance gene was thereby introduced in the reverse orientation respective to the *Fech* sequences (Fig. 1). (4) Mouse genomic DNA fragments of ∼6 kbp containing the flanking parts of the *Fech* gene including exons 2 and 5 (obtained by PCR from cosmid clones) were then joined to both sides of this intermediate assembly to serve as homology arms for recombination. Details of the cloning procedure are available on request.

After validation by sequencing and restriction digestion, the targeting construct was linearized with *Pvu*I and transfected into the C57BL/6 mouse Bruce4 embryonic stem (ES) cell line (Köntgen et al., 1993). After selection for growth in the presence of neomycin, homologous recombinant ES cell clones were identified by Southern blot. Moreover, total RNAs from ES cells containing the correct integration of the human insert, genotype wt/C (clone II-1C1), as well as from control ES cells (B4) were analyzed by RT-qPCR. In particular, the aberrant splicing product (63 bp insertion between exons 3 and 4) was detected, and the intensity of the fluorescence signal was proportional to the amount of cDNA in the reaction (data not shown). Sequencing of cloned RT-PCR products from II-1C1 cells revealed the correctly spliced mouse mRNA and the chimeric mRNA, as well as the aberrant splicing product (data not shown). In addition, mRNAs with a skipping of exon 3 were detected (Fig. S3).

ES cells from the positive clone II-1C1 were then injected into BALB/c hybrid blastocysts. Male chimeric mice were obtained and crossed to C57BL/6J females to establish heterozygous germline offspring carrying the humanized *Fech* allele on a pure C57BL/6J background. The neomycin resistance cassette was then removed by crossing with an OzCre mouse expressing Cre recombinase from the Gt(ROSA)26Sor locus on a C57BL/6J background. The new mouse line in a C57BL/6J background has been registered with the designation C57BL/6J-Fech*^Tm1(FECH)Emi^*. The humanized c.315-48C allele will be referred to as Emi hereafter. In these mice, we confirmed the correct integration of the human sequences by amplifying exons 3 and 4 with surrounding intronic sequences from genomic DNA of an Emi/wt (C57BL/6J) mouse and sequencing of these PCR products (Fig. S5 and Fig. S6, respectively).

### Breeding properties of mice carrying the c.315-48C *FECH* allele

By intercrossing heterozygous Emi/wt mice (C57BL/6J-Fech*^Tm1(FECH)Emi^* line), we consistently obtained wt/wt and Emi/wt pups in a ratio of 1:1.75 (Table 1). Importantly, no live Emi/Emi mice were ever born. Upon dissection of pregnant females, Emi/Emi embryos could be detected at embryonic day (E)11, at which time they looked smaller – similar to embryos of the other genotypes 1-2 days previously. At E13, only remnants of Emi/Emi embryos were detected within their placental structures.

**Table 1.**
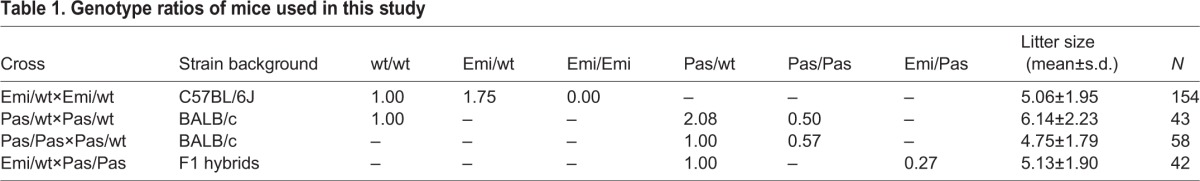
**Genotype ratios of mice used in this study**

Because of the inability to breed Emi/Emi mice and in order to mimic the genotype of the vast majority of human EPP patients, we decided to cross Emi/wt mice with homozygous animals of the *Fech* mutant line m1Pas (C.Cg-Fech*^m1Pas^*/J) (Boulechfar et al., 1993; Tutois et al., 1991). This allele will be referred to as Pas for short. Such a cross produces F1 hybrids between C57BL/6J and BALB/c/J that are genetically identical except for differences in the *Fech* gene. The ratio between the two genotypes wt/Pas and Emi/Pas has so far been 1:0.27 (Table 1), which is considerably lower than the expected 1:1 ratio. In comparison, the birth frequency of Pas/Pas homozygotes of the C.Cg-Fech*^m1Pas^*/J line under our breeding conditions has been 0.5 of the expected 25% in Pas/wt×Pas/wt crosses and 0.57 of the expected 50% in Pas/Pas×Pas/wt crosses (Table 1), Emi/Emi and Emi/Pas animals are therefore subject to a higher prenatal mortality rate than Pas/Pas animals.

### Emi mice reproduce the alternative splicing associated with the human c.315-48C allele but also skip exon 3 in the majority of their *Fech* transcripts

**Fig. 2.**
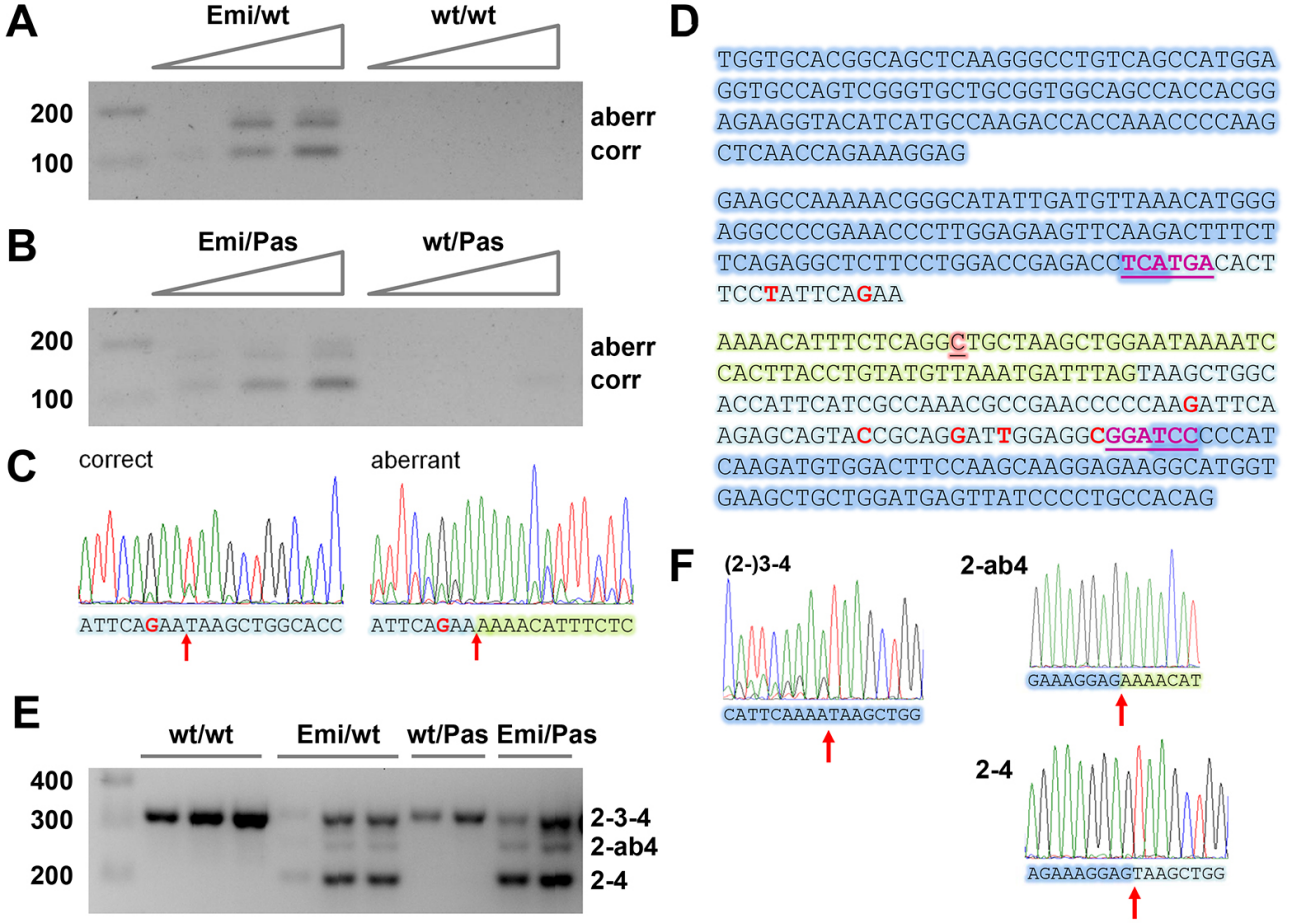
**Alternative splicing of transcripts from the humanized *Fech* allele in transgenic mice.** (A,B) RT-PCR analysis (40 cycles) with primers specific for the humanized allele of increasing amounts of total liver RNA from (A) Emi/wt and wt/wt C57BL/6J mice and (B) Emi/Pas and wt/Pas F1 hybrid mice. Both correctly and aberrantly spliced transcripts are detected. A band migrating slightly above the aberrant one is due to heterodimer formation. (C) Sanger sequence traces of PCR-amplified cDNA corresponding to correct and aberrant transcripts. The splice junctions are marked by a red arrow. (D) Sequence of exons 2 to 4 of the humanized allele. The shading indicates mouse exon parts (dark blue), human exon parts (light blue) and the extra 63 nucleotides added as a result of aberrant splicing (light green). Within the exons, single nucleotide differences between human and mouse are marked in red (all silent changes not affecting the protein sequence). The c.315-48C polymorphism is highlighted in red and underlined. Magenta, underlined letters: *Bsp*HI and *Bam*HI sites in exons 3 and 4. (E) RT-PCR analysis (35 cycles) with primers in the common parts of exons 2 and 4 of total spleen RNA from multiple mice of the indicated genotypes. (F) Sanger sequence traces demonstrating that the bands correspond to full-length *Fech* mRNA [(2-)3-4], as well as exon 3-skipped mRNA spliced to the aberrant and correct 3′ ss (2-ab4 and 2-4, respectively). For primer sequences, see Table S1.

To analyze the transcripts of the humanized *Fech* (Emi) allele, we subjected liver cDNA from Emi/wt (C57BL/6J) and Emi/Pas (F1 hybrid) mice as well as from corresponding control mice that lack the Emi allele to RT-PCR analysis. An amplification with primers corresponding to the humanized parts of exons 3 and 4, which contain several mismatches to the mouse sequence, generated products only in cDNA of mice carrying the Emi allele (Fig. 2A,B). Signals corresponding to correctly spliced and mis-spliced RNA were obtained. These RNAs seemed to be present in very low amounts, as the PCR products increased with each increment in cDNA input, even though the PCR had been performed for 40 cycles which should be saturating for abundant transcripts. Further evidence for a low abundance of Emi-specific transcripts came from our attempts to use these and other primers to quantify both transcript species by quantitative RT-PCR. Both were amplified with Ct values of ≥35 cycles (i.e. close to background) which has so far precluded their reliable quantification (data not shown). To confirm the splice junctions, we used spleen cDNA from an Emi/wt (C57BL/6J) mouse to prepare PCR products extending from the humanized part of exon 3 to exon 5 and corresponding to correctly and aberrantly spliced Emi-specific transcripts. Direct sequencing of these PCR products confirmed the expected splicing (Fig. 2C; 2D shows the sequences of the exons 2 to 4).

Because preliminary experiments in the II-1C1 ES cells used to generate the Emi mice had indicated that some of the Emi-derived RNAs undergo exon 3 skipping, we analyzed total transcripts arising from all *Fech* alleles by using PCR primers binding to common parts of exons 2 and 4. This revealed that all mice carrying the Emi allele produce both exon 3-containing and exon 3-skipped RNA (Fig. 2E; confirmed by sequencing in Fig. 2F). In contrast, we could not detect any exon 3 skipping in mice that contain combinations of wt and Pas *Fech* alleles (Fig. 2E) or in immortalized lymphoblasts from a human individual with two c.315-48C alleles (Fig. S4). Considering that shorter amplicons tend to be replicated more efficiently than longer ones, the exon 3-containing and -lacking RNAs appear to be present in Emi/wt or Emi/Pas mice in approximately equal amounts. Moreover, a band corresponding to longer, aberrantly spliced but exon 3-containing, Emi transcripts could not be detected on such gels. This implies that only a very small fraction of Emi-derived RNAs contains exon 3 that is either correctly or aberrantly spliced to exon 4, whereas the majority lacks exon 3. In contrast, there is a weaker band for full-length and exon 3-skipped RNAs of mice carrying the Emi allele, which corresponds in size and sequence to a splicing product linking exon 2 to the aberrant 3′ ss upstream of exon 4 (Fig. 2E,F).

### Morphological features, tissue and blood parameters of Emi/Pas mice

**Fig. 3.**
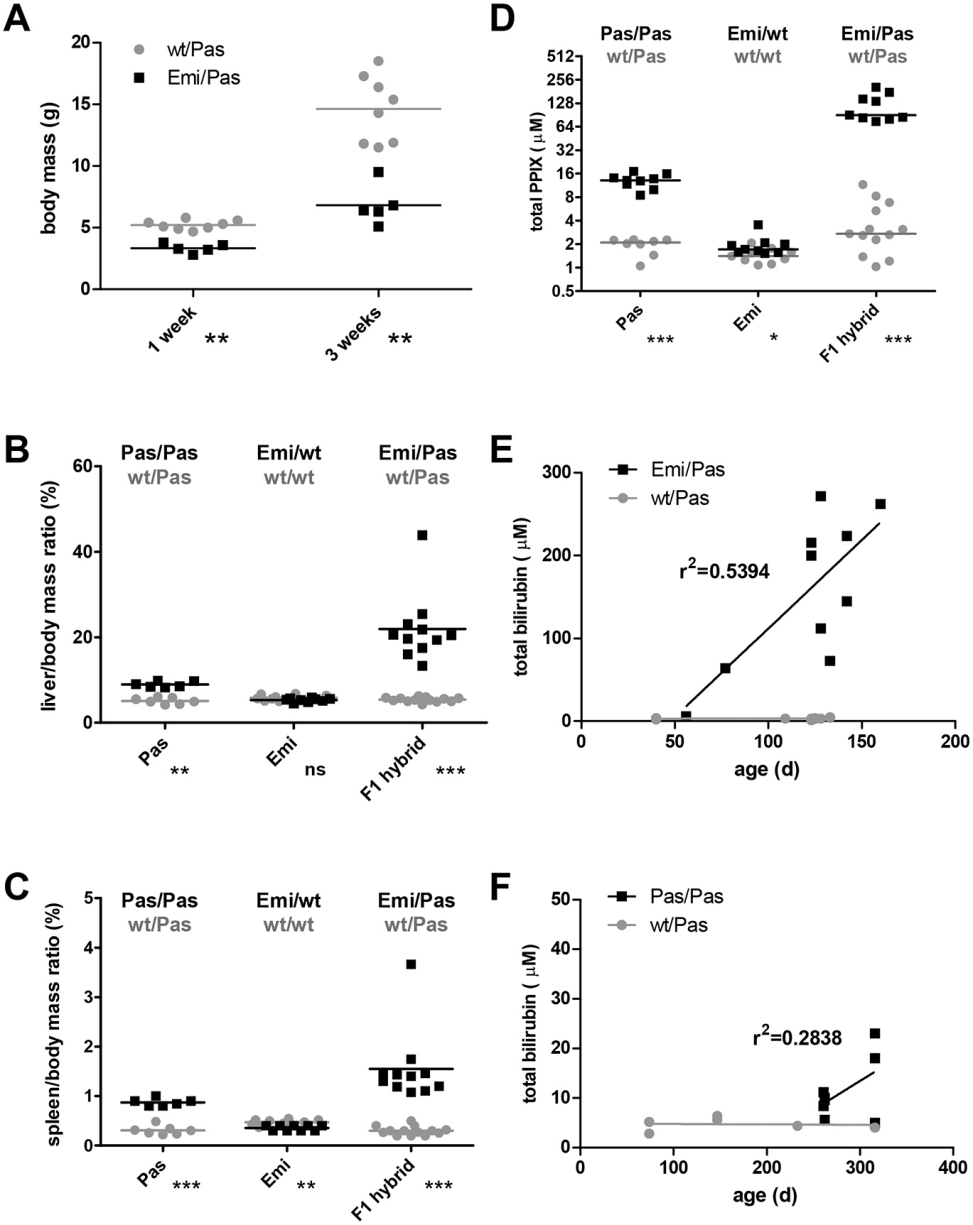
**Growth, organ mass and biochemical parameters of EPP mouse models.** (A) Total body mass of Emi/Pas and wt/Pas F1 hybrid mice. Liver (B) and spleen (C) masses relative to total body mass. (D) Total protoporphyrin IX concentration in EDTA-anticoagulated blood samples. Pas, C.Cg-Fech*^m1Pas^*/ J line; Emi, C57BL/6J-Fech*^Tm1(FECH)Emi^* line; F1 hybrid, cross between Emi/wt (C57BL/6) and Pas/Pas (BALB/c). *P*-values of two-tailed Mann–Whitney *t*-test are indicated: ns, *P*>0.05; *P<0.05; **P<0.01; ***P<0.001. (E,F) Plasma bilirubin concentration as a function of age for F1 hybrid (E) and Pas (F) mice.

The most salient feature of Emi/Pas F1 hybrid mice compared with their healthy Pas/wt littermates, is a retarded growth and reduced body size and mass. This difference is small but already significant shortly after birth, and it becomes more pronounced with age (Fig. 3A). Moreover, the livers and spleens of these mice are enlarged. These features are most apparent when the organ mass is displayed in relation to body mass (Fig. 3B,C). An elevated liver and spleen mass was also measured in our Pas/Pas (BALB/c/J) mice, in agreement with published data (Tutois et al., 1991). Importantly, the increase in liver and spleen mass was more pronounced in Emi/Pas F1 hybrids than in Pas/Pas homozygous mice. Amounting to ∼20% of the body mass, the liver of Emi/Pas F1 animals was so big that it often became perceivable by an enlarged abdomen. The liver tissue also appeared more rigid upon palpation compared with the usual soft texture.

**Fig. 4.**
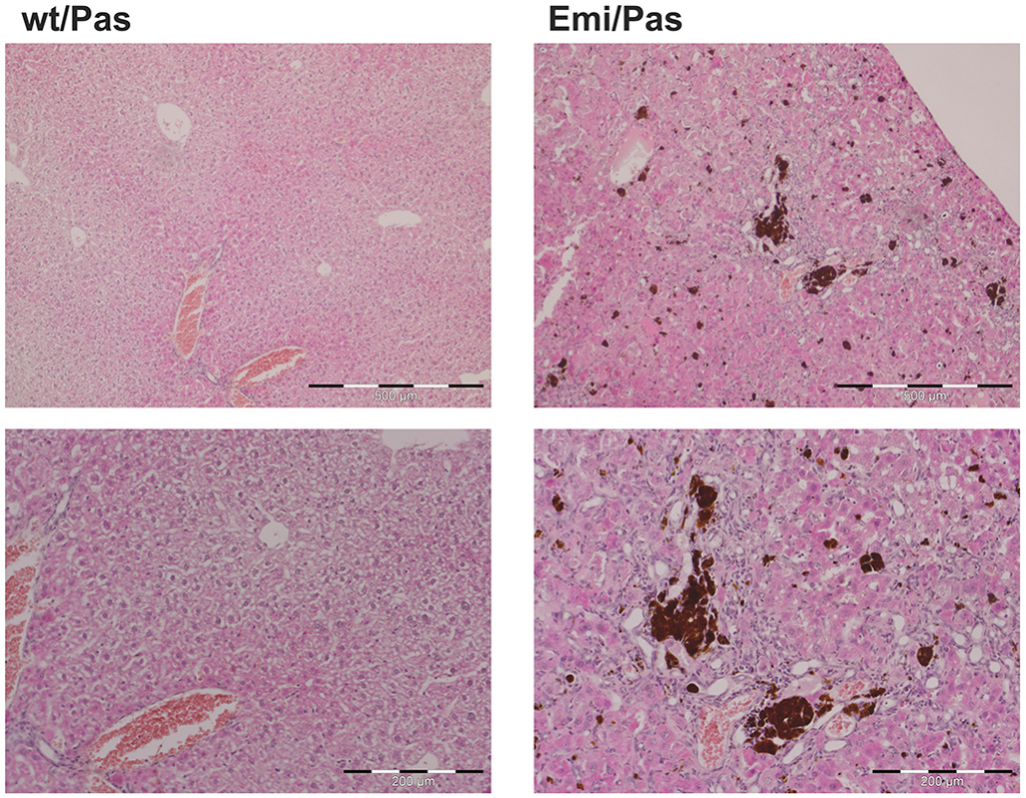
**Liver histology in EPP mouse model.** Representative images of H&E-stained liver sections of wt/Pas and Emi/Pas F1 hybrid mice at two different magnifications. Note the presence of small cells (presumably macrophages) and brown inclusions (presumably PPIX deposits) in the Emi/Pas mouse. Scale bars: 500 µm (top) and 200 µm (bottom).

Microscopy analysis of Hematoxylin and Eosin-stained liver sections revealed a pronounced hepatobiliary pathology in livers of Emi/Pas F1 hybrids (Fig. 4). There was an increase in small cells (presumably macrophages). Most prominently, many brown pigment granules were present in biliary canaliculi and portal macrophages. As they could not be stained with Prussian Blue (data not shown), these granules probably represent PPIX deposits, as observed in human EPP patients with a pronounced liver pathology (Lecha et al., 2009).

Biochemical analyses of blood samples revealed that Emi/Pas F1 hybrids have extremely high levels of PPIX (Fig. 3D). The values were 6- to 8-times higher than in Pas/Pas (BALB/c) mice, which also had elevated PPIX levels as expected. Additionally, most of the Emi/Pas F1 hybrids showed elevated serum bilirubin levels that tended to increase with age (Fig. 3E). Even though this was also the case for some of the Pas/Pas (BALB/c) mice (Fig. 3F), the levels reached in Emi/Pas F1 hybrids were higher and the increase seemed to occur earlier than in Pas/Pas animals. In such animals, jaundice was also evident during post-mortem inspection by a yellowish taint of the dermis, muscles and internal organs. In contrast, the serum levels of the liver enzymes alanine aminotransferase (ALT) and aspartate aminotransferase (AST) were not significantly different between the various genotypes (data not shown). We also could not detect any significant differences in serum iron concentration (data not shown).

Hematological indices revealed that Emi/Pas F1 hybrids have a pronounced microcytic anemia. Red blood cell counts as well as hematocrit, mean corpuscle (cell) volume (MCV), mean corpuscular hemoglobin (MCH) and mean corpuscular hemoglobin concentration (MCHC) were reduced (Table 2). Moreover, the numbers of reticulocytes were elevated, presumably to compensate for the lack of mature erythrocytes. Interestingly, the number of platelets increased and eosinophils decreased in Emi/Pas F1 animals. In contrast, we did not observe any significant differences in blood cell composition between Emi/wt and wt/wt mice of the C57BL/6J strain (data not shown).

**Table 2.**
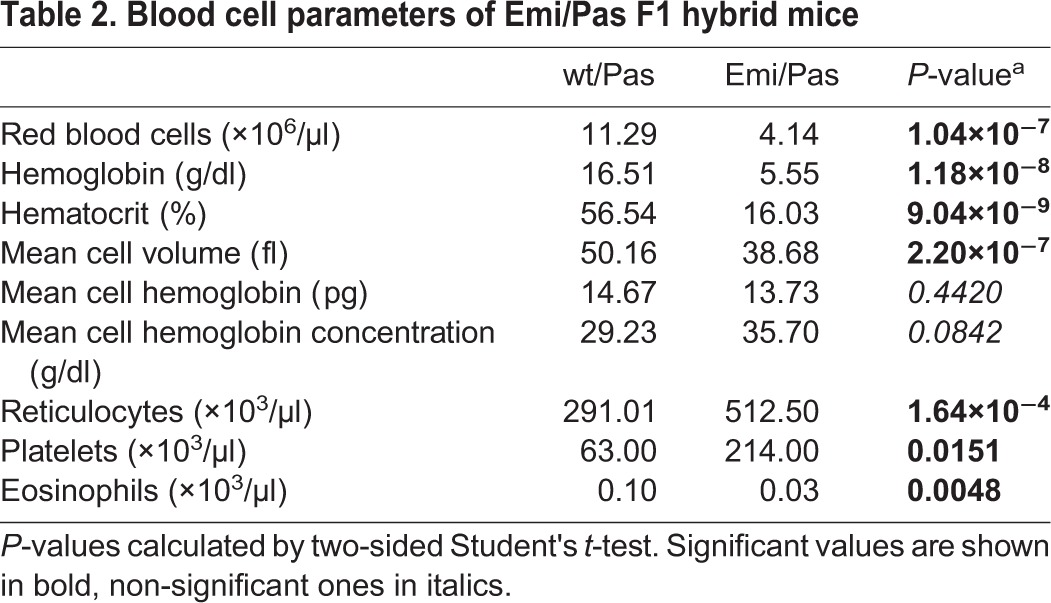
**Blood cell parameters of Emi/Pas F1 hybrid mice**

### Light sensitivity

**Fig. 5.**
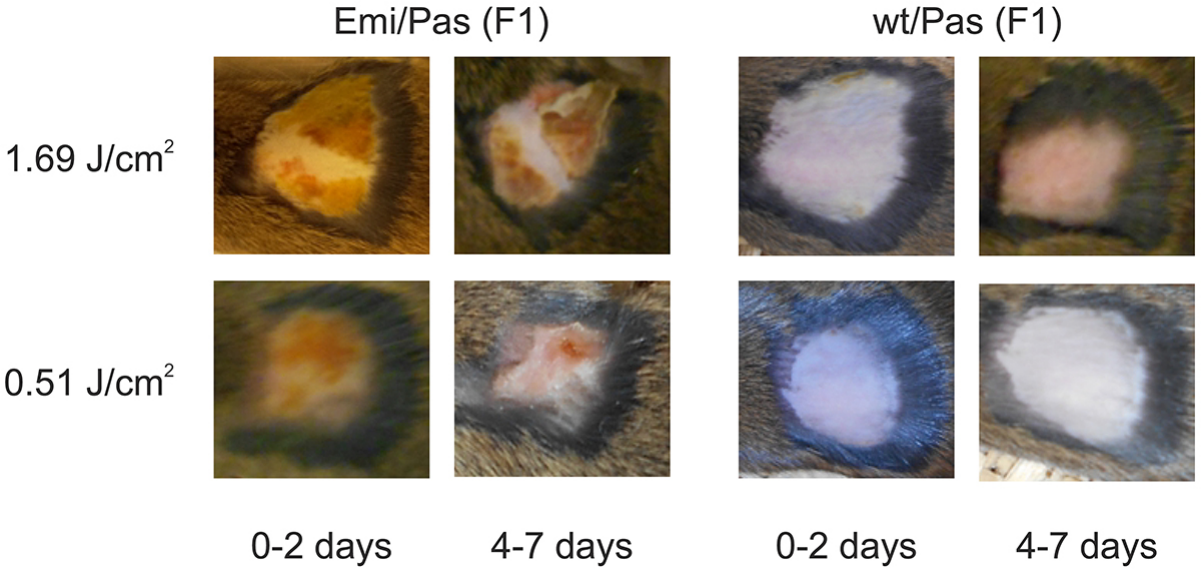
**Skin photosensitivity tests in EPP mouse model.** Areas of 1-3 cm^2^ on the back of F1 hybrid Emi/Pas and wt/Pas mice were shaved and epilated, and exposed to light doses indicated on the left. Photographs were taken at different times post-exposure to document visible skin reactions and healing. The Emi/Pas mice show erythema and an orange exudate at both light doses, which forms a scab or small ulcerations in the process of healing by 4-7 days after exposure.

Skin photosensitivity is the main clinical symptom of patients suffering from EPP. To assess the degree of skin sensitivity of the new EPP mouse model, we exposed small areas (1-3 cm^2^) of shaved skin to various doses of 400-420 nm light. As expected, we did not observe any severe effect of light on Pas/wt F1-hybrid control mice. The only exception were two small lesions on one animal treated with the highest light dose (data not shown) which might, however, have been caused by the shaving. In sharp contrast, all tested light doses elicited acute symptoms on the exposed skin areas of Emi/Pas mice. Erythema and an orange exudate (which dried within 24 h) became visible by 2-3 h post-exposure. Additionally, we observed behavioral changes indicating pain. At doses of 1.69-2.3 J/cm^2^ (∼10 min exposure under our experimental conditions), we recorded severe symptoms such as a hunched position, reduced movement, occasional shivering, closed eyes and drawn back ears that required both systemic and local analgesic treatment. At doses of 0.51-0.68 J/cm^2^ (∼3 min exposure), the pain symptoms were more moderate: the mice were frequently twitching and tried to lick or scratch the light-exposed areas. In these cases, local analgesia with cream was sufficient to control the pain. These pain symptoms disappeared by 24 h post-exposure, but the morphological alterations persisted for several days. After 4-7 days, the dried exudate started to peel off. More strongly affected areas were covered with a scab or showed small ulcerations in the process of healing (Fig. 5). This light sensitivity of Emi/Pas F1 hybrids was considerably stronger than that of Pas/Pas (BALB/c) animals tested under the same conditions. Pas/Pas mice required light doses in excess of 6 J/cm^2^ to produce similar skin alterations and generally showed less-pronounced pain symptoms.

## DISCUSSION

We have generated the first EPP mouse model that represents the human inheritance pattern which was originally referred to as an autosomal dominant trait with incomplete penetrance. In the 1980s, Went and Klasen put forward a three-allele hypothesis to explain the EPP phenotype as being the result of an interaction between wild-type and mutated *FECH* alleles and a third, yet unknown, disease-causing allele (Went and Klasen, 1984). It was not until 2002 that the nature of the disease-causing ‘third allele’ was identified as a splice-modulating single nucleotide polymorphism in intron 3 of the *FECH* gene (c.315-48C) (Gouya et al., 2006, 2002). The combination of this allele with a debilitating *FECH* mutation is the predominant genetic cause of EPP. The availability of a mouse model for this inheritance pattern is thus an important prerequisite for pre-clinical testing of EPP therapies.

Our data demonstrate that both correct and aberrant splicing of the human *FECH* intron 3 carrying the splice-modulating c.315-48C polymorphism (Emi allele) do occur in the mouse background and that the aberrant splice product is identical to that in humans (Fig. 2A-C). However, the humanized *Fech* gene also undergoes a second kind of alternative splicing – the skipping of exon 3 (Fig. 2E,F). Additionally, a small amount of RNA gets spliced from exon 2 to the aberrant 3′ ss generated by c.315-48C. Skipping of exon 3 is in fact the predominant event, leaving only a very small proportion of transcripts that undergo splicing from exon 3 to the aberrant and correct splice acceptor sites of exon 4. The RNA that correctly joins exons 2 and 4 appears to be stable, since it accumulates in approximately equal amounts as the full-length transcripts that are produced by the other (wt or Pas) allele. Its stability is expected, because the non-inclusion of 120 nucleotides present in exon 3 retains the translational reading frame, so that the RNA will not be subjected to NMD. Exon 3 encodes 40 amino acids from the N-terminus of the mature protein. Based on the structure of human ferrochelatase (Wu et al., 2001), deletion of this part should render the protein non-functional. However, the altered protein might also be eliminated because of mis-folding, instability and/or might fail to get imported into mitochondria.

These findings underscore the difficulty to reproduce human splicing mutations correctly in a different species such as the mouse. Despite the fact that certain human splicing mutations have been reproduced successfully (Gladman et al., 2010; Lewis et al., 1998), engineering humanized genes in mice can lead to unforeseeable splicing outcomes, as observed by Garanto et al. (2013) in their attempt to produce a model for Leber congenital amaurosis. In their case, a new aberrant exon was inserted into *Cep290* transcripts.

Our finding that the Emi allele produces only very small amounts of correctly spliced, exon 3-containing RNA that can be translated into functional ferrochelatase explains the severity of the observed phenotype. In contrast to human homozygous carriers of the splice-modulating c.315-48C polymorphism who are asymptomatic or, in rare cases, slightly symptomatic (Gouya et al., 2002; Schneider-Yin et al., 2008), homozygous Emi/Emi (C57BL/6J) embryos die *in utero* around day E12. Moreover, the Emi/Pas F1 hybrid mice present a more severe EPP phenotype than Pas/Pas (BALB/c/J) homozygotes. This is reflected in the reduced survival up to birth (0.27 compared with ∼0.55; Table 1), growth retardation, higher PPIX levels (Fig. 3), impaired hematological parameters (Table 2) and the early onset of bilirubinemia and liver abnormalities (Fig. 3). Since the Pas allele has been reported to produce ferrochelatase with an activity of ∼6% of the wild-type enzyme (Boulechfar et al., 1993), we assume that the amount of functional FECH enzyme produced by the Emi allele is below this level. Finally, even in heterozygous carriers of the Emi allele, which are asymptomatic for most of the assessed parameters, we detected small significant changes in relative spleen size and PPIX levels (Fig. 3C,D).

Despite the additional exon 3 skipping and the pronounced symptomatic severity, the newly created Emi/Pas mouse model reproduces important signs and symptoms of the human condition. The most prominent symptom of human EPP, the very painful light sensitivity, is faithfully reproduced in the Emi/Pas mouse model (Fig. 5). In fact, the light sensitivity may be similar or even stronger than that observed in human patients, as an exposure of only ∼3 min produced marked behavioral reactions and subsequent skin lesions. The sensitivity of Emi/Pas F1 hybrid mice was at least three times stronger than that manifested by Pas/Pas (BALB/c/J) mice (requiring a 3.3-times shorter exposure while showing stronger symptoms). However, these symptoms were only observed when the skin area was closely shaved/epilated prior to exposure. Importantly, under our normal housing/lighting conditions, neither Emi/Pas nor Pas/Pas mice displayed any pain symptoms or developed reactions of fur-free areas, such as ears, nose, feet or tail. This may vary from one mouse facility to another, as skin lesions have previously been reported to occur under normal housing and lighting conditions in the less light-sensitive Pas/Pas mice (Tutois et al., 1991).

The Emi/Pas mouse model also exhibits a microcytic, hypochromic anemia with lower hemoglobin, hematocrit, mean corpuscular volume and mean cell hemoglobin, as can be expected from a deficiency of heme biosynthesis. This anemia seen in Emi/Pas mice is stronger than that observed in Pas/Pas mice (Tutois et al., 1991) or in most EPP patients where the anemia is usually not clinically relevant (Holme et al., 2007). In Emi/Pas mice, the hemoglobin levels, hematocrits and red blood cell counts were all reduced to 30-40% of the levels seen in wt/Pas control mice (which lay in the normal range for BALB/c or C57BL/6 mice). Additionally, reticulocytes were slightly elevated. Iron levels were not significantly different between Emi/Pas and wt/Pas mice (data not shown). However, as the erythropoiesis in mice differs from the human, the direct comparison of the influence on hematological parameters has to be further validated.

The increase in liver and spleen size are signs of a severe liver injury, as seen in a minority (∼3.75%) of EPP patients (Anstey and Hift, 2007; Doss and Frank, 1989; Rademakers et al., 1990). Apparently, the liver injury in our Emi/Pas mouse model is purely cholestatic as only bilirubin, not the transaminases, are elevated. This cholestatic liver involvement results in the discoloration of the liver and in deposits of brown pigments, presumably consisting of protoporphyrin IX crystals. Hepatic protoporphyrin deposits are typical in EPP-related liver damage and also occur in Pas/Pas mice of older age (Abitbol et al., 2005; Tutois et al., 1991). However, the liver symptoms of Emi/Pas mice are again stronger than those of Pas/Pas mice, as seen by the strong and early increase in bilirubin levels compared with the more moderate rise in older Pas/Pas mice (Fig. 3E).

The strong contribution of exon 3 skipping to the phenotype of Emi/Pas mice has consequences for the future use of this animal model. The *in vivo* efficiency of therapeutic approaches targeting the aberrant splicing process between exons 3 and 4 can be tested by a specific RT-PCR assay, as shown in Fig. 2A,B; however, even if they are successful, these approaches will probably not have a big impact on clinical parameters. Possible solutions to this problem are the creation of a more refined model that does not show exon 3 skipping or a simultaneous treatment that reduces this skipping along with one correcting the aberrant splicing between exons 3 and 4.

However, this humanized model may also provide a useful experimental system to study other questions related to EPP such as EPP-related liver disease, novel treatment options for the intense pain caused by light exposure (Babes et al., 2016) or further investigations of the aberrant splicing itself. As previously shown in cell culture studies, the alternative 3′ ss at position -63 of intron 3 appears to have a regulatory function, because it is regulated by the availability of iron via the iron-, oxygen- and 2-oxoglutarate-dependent dioxygenase JMJD6 (Barman-Aksözen et al., 2013). Emi mice could be crossed with (conditional) knockout strains for JMJD6 and/or its target, U2AF (also known as ZRSR1) or yet other potentially involved gene products to investigate the influence of these factors on splicing regulation of this human intron that plays such a pivotal role in EPP.

## MATERIALS AND METHODS

### Cell lines

EBV-transformed lymphoblasts of non-porphyric human subjects homozygous for the c.315-48C and c.315-48T allele, respectively, have been described previously (Barman-Aksözen et al., 2013). Bruce4 is a C57BL/6 mouse embryonic stem (ES) cell line used by Ozgene (Bentley, WA, Australia) for homologous integration purposes (Köntgen et al., 1993). The cell lines are checked for mycoplasma contamination on a regular basis.

### Mouse breeding and genotyping

The Emi strain containing a partly humanized *Fech* gene with the c.315-48C variation (C57BL/6J-Fech*^Tm1(FECH)Emi^*) was generated by one of our groups (J.B., X.S. and E.M.) in collaboration with Ozgene as described in the first Results section. The *Fech* mutant strain m1Pas (Tutois et al., 1991) was obtained after cryo-recovery, from Jackson Laboratories (Bar Harbor, ME, USA; C.Cg-*Fech^m1Pas^*/J, 002662). F1 hybrids were generated by crossing Emi/wt and Pas/Pas animals of these two lines. These F1 offspring were not used for further breeding.

All mice were kept in individually ventilated cages under specific-pathogen-free (SPF) conditions at the central animal facility of the University of Berne, Switzerland. Animals affected by EPP (Pas/Pas and Pas/Emi) were kept in the lower rows of cage racks but without further light protection. Under normal lighting conditions in our facility (12 h:12 h light:dark cycles), these EPP mice did not develop any skin irritations. All procedures involving animals were performed in accordance with Swiss animal protection law and according to our specific permission BE92/13.

Genomic DNA was isolated from toe clip biopsies, taken 1 week after birth, by using the KAPA mouse genotyping kit (Kapa Biosystems, Wilmington, MA, USA), according to the manufacturer's protocol. For the PCR reactions, 25 μl of reactants containing 1 μl extracted DNA, 500 nM of the corresponding primers, 1.6 μM MgCl_2_ in 1× KAPA mix were incubated for 15 s at 94°C, 15 s at 60°C, and 30 s at 72°C for 35 cycles. The mouse *Fech* gene was amplified with primers binding upstream and downstream of exon 3, respectively, which yield an amplicon of 313 bp. To detect the Pas point mutation, which destroys the *Bsp*HI site in exon 3 (Boulechfar et al., 1993), half of such a completed PCR reaction was digested with *Bsp*HI and compared with undigested material by agarose gel electrophoresis. The humanized Emi allele was detected in a PCR containing the two mouse primers and a third, human-specific reverse primer that yields an amplicon of 271 bp with the mouse forward primer. Additional amplifications with primers specific for the *Sry* locus served to identify the sex of the animals (Meyer et al., 2009). Primer sequences are listed in Table S1.

### Collection of blood and tissue samples

Mice were sacrificed by CO_2_ inhalation and cervical dislocation. The masses of the entire body, liver and spleen were determined. Blood samples were collected from the heart with glass capillaries and transferred into EDTA- and heparin-coated tubes (MiniCollect Heparin or EDTA, Greiner Bio-One, St Gallen, Switzerland). Livers and spleens were harvested for further histological and RNA analysis.

### Biochemistry and hematology of blood samples

Hematological analysis was performed in a IDEXX ProCyte Dx Hematology Analyzer. Metal-free and zinc-bound protoporphyrin IX were quantified in 50 µl EDTA-anticoagulated blood samples as described by Rossi and Garcia-Webb (1968) with slight modifications. Total bilirubin was measured in plasma samples by using the Bilirubin Total Gen.3 kit on an automatic Analyzer Cobas c501 (Roche Diagnostics, Rotkreuz, Switzerland). Transaminases ALT and AST were measured in the same samples by using the ALTL and the ASTL kits, respectively, on Cobas c501. All samples were diluted 1:4 with the Diluent Universal (Roche Diagnostics, Rotkreuz, Switzerland) prior to the measurement in order to reach the minimal volume required by the analyzer.

### Histology

Liver samples were fixed with 4% paraformaldehyde (Sigma-Aldrich) for 24 h and embedded in paraffin. Sections were cut at 5 µm with a microtome (Leica, Reichter-Jung, Supercut 2050) followed by staining with H&E according to standard procedures.

### RNA extraction and analysis

Mouse tissue samples from liver and spleen (20-30 mm^3^) were snap frozen and crushed with a pestle (BioConcept 100539). RNA was isolated in 1 ml Trizol reagent (0.8 M guanidine thiocyanate, 0.4 M ammonium thiocyanate, 0.1 M sodium acetate pH 5.0, 5% v/v glycerol, 38% v/v saturated acidic phenol, 5 mM EDTA, 0.5% sodium lauroylsarcosine, in diethylpyrocarbonate-treated water). The samples were vortexed vigorously and kept for 5 min on ice. Then 200 µl chloroform was added, thoroughly mixed and, after an additional incubation of 5 min, centrifuged for 15 min at 12,000 ***g***. The aqueous phase was collected, and RNA was precipitated by addition of 600 µl of isopropanol for 10 min at room temperature. The precipitated RNA was centrifuged for 15 min at 12,000 ***g*** in 4°C, and the pellet was washed with 80% ethanol, centrifuged for 5 min at 16,100 ***g***, air-dried and resuspended in RNase-free water.

### Light-sensitivity tests

Small areas (1-3 cm^2^) on the backs of mice (always Pas/wt and Pas/Emi together) were shaved and epilated with VEET cream. One day later, the shaved skin area was exposed to light from a Megaman Plant Lamp (BR0515P) for 3 or 10 min at a dose of 0.51-0.68 J/cm^2^ or 1.69-2.3 J/cm^2^, respectively. The UV-B and UV-C light was filtered by a glass placed between the mouse and the lamp to avoid burns. Pictures were taken at day 1 post-exposure and at later times to document the occurrence and healing of skin symptoms. Pain symptoms were recorded by observation and in some cases documented by video recording.
